# What Can Canada Learn From Accountable Care Organizations: A Comparative Policy Analysis

**DOI:** 10.5334/ijic.5677

**Published:** 2022-04-01

**Authors:** Allie Peckham, David Rudoler, Dominika Bhatia, Sara Allin, Reham Abdelhalim, Gregory P. Marchildon

**Affiliations:** 1 Center for Innovation in Healthy and Resilient Aging, Edson College of Nursing and Health Innovation, Arizona State University, 550 North 3rd St, Phoenix, Arizona, 85004, USA; 2 North American Observatory on Health Systems and Policies, 155 College St, Suite 425, Toronto, Ontario M5T 3M6, Canada; 3 Institute of Health Policy, Management and Evaluation, University of Toronto, 155 College St, Suite 425, Toronto, Ontario M5T 3M6, Canada; 4 Faculty of Health Sciences, University of Ontario Institute of Technology, 2000 Simcoe St N, Unit UA3000, Oshawa, Ontario L1H 7K4, Canada; 5 Institute for Mental Health Policy Research, Centre for Addiction and Mental Health, 33 Russell St, Toronto, Ontario M5S 2S1, Canada

**Keywords:** Accountable care organizations, comparative policy analysis, integrated care models, narrative review, Canada, United States

## Abstract

**Introduction:**

Accountable Care Organizations (ACOs), implemented in the United States (US), aim to reduce costs and integrate care by aligning incentives among providers and payers. Canadian governments are interested adopting such models to integrate care, though comparative studies assessing the applicability and transferability of ACOs in Canada are lacking. In this comparative study, we performed a narrative literature review to examine how Canadian health systems could support ACO models.

**Methods:**

We reviewed empirical studies (published 2011–2020) that evaluated ACO impacts in the US. Thematic analysis and critical appraisal were performed to identify factors associated with positive ACO impacts. These factors were compared with the Canadian context to assess the applicability and transferability of ACO models within Canada.

**Findings:**

Physician-led models, global budgets and financial incentives, and focus on collaborative care may optimize ACO impacts. While reforms towards alternative payments and team-based care are not unprecedented in Canada, significant further reforms to physician remuneration, intersectoral collaboration, and accountability for performance are required to support ACO-like models.

**Conclusion:**

This comparative study uncovered several insights on the applicability and transferability of ACOs to the Canadian context. Further comparative research outside the US is needed to infer the essential components of successful ACO models.

## Introduction

The Accountable Care Organization (ACO) model, which was implemented in the United States (US) under the Patient Protection and Affordable Care Act, 2010, aims to achieve reduced costs and coordinate care by aligning incentives among providers and payers [1]. These pursuits are not new or unique in high-income countries. Over the past decade, there has been growing awareness of the deficiencies of fragmented, poorly integrated health systems [2,3,4]. In Canada, the 2002 federal report offered a “Renewed Vision” for Canada’s health system [5]. Beyond the goals of integration and coordination, financial sustainability was a top concern. Similar to the intended outcomes of ACOs, there has been increasing interest in achieving integration and the “Quadruple-Aim” of improving patient and caregiver experience, improving population health, improving healthcare provider experience, and keeping per capita costs sustainable [1].

While there is no unifying definition of integrated care, it is often contraposed to siloed, fragmented, and episodic care [6,7]. The ACO model is recognized as a model of integrated care that aligns with the “process-based” definition of integrated care described by the World Health Organization, where “integration is a coherent set of methods and models on the funding, administrative, organizational, service delivery and clinical levels designed to create connectivity, alignment and collaboration within and between the cure and care sectors” [4]. Goals of integrated care are to enhance the quality of care and life, improve consumer satisfaction and improve system efficiency [6,7]. These goals match those of ACO models in improving the “Quadruple-Aim” outcomes.

The implementation of ACOs can be seen as one of the most recent attempts to move health systems toward achieving integrated care and the “Quadruple-Aim” outcomes, and Canadian and international governments have shown an increasing interest in the ACO model following experiences with ACOs in the US. For example, in 2018, the NHS in England announced a plan to redesign care and introduce ACOs [8]. After a public outcry, these plans were changed to Integrated Care Organizations or ICOs, which describe a rage of care models that attempt to link multiple organizations to a single organization responsible for integration [8,9,10,11].

Yet, a range of opinions remain about whether ACOs or ACO-like models are the most effective way to achieve improvements in sustainability and integration [10,12,13,14]. In addition, there is limited understanding of the core attributes of ACOs that contribute to their (potential) impact and the extent to which the ACO model can be transferred across jurisdictions. The successful translation of policies and programs is a core challenge in major reform efforts, as it not only requires an in-depth understanding of the intervention and the evaluative evidence, but also the context within which the intervention will be implemented [15].

To address this knowledge gap, we conducted a narrative review, thematic analysis, and quality appraisal of the scientific and grey literature to identify the attributes of ACOs that are closely associated with positive impacts. We then consider whether the most prominent factors associated with positive impacts can be adopted in the Canadian healthcare context.

## Methods

### Review of Scientific and Grey Literature

We used a combination of structured and iterative search techniques to identify empirical quantitative, qualitative, and mixed methods peer-reviewed publications and grey sources that evaluated the impact of ACOs on any of the Quadruple-Aim outcomes between January 2011 and December 2020. We searched MEDLINE (Ovid), the Centers for Medicare and Medicaid Services (CMS) websites, Google/Google Scholar, and McMaster University’s Health Systems Evidence Service using terms related to ACOs and evaluative studies, predictors of success, and Quadruple Aim outcomes. We then searched forward and backward citations, including reference lists of two review articles [16,17] to obtain additional primary studies. A total of 63 publications on ACOs were identified.

### Analysis

The analysis involved two stages. First, two researchers (AP, DB) developed and piloted a data extraction form that classified the following elements of all selected articles: research methods, type of ACO model studied, and the main evaluative findings presented. Two researchers (DB, RA) then extracted the relevant information from the literature. Second, we used an inductive approach to identify factors associated with positive impact. One researcher (DB) selected 27 articles where ACOs were shown to have a positive impact on at least one of the Quadruple-Aim outcomes using specifically qualitative, quantitative, or mixed methods data. Two researchers (AP, RA) then inductively and thematically assessed the extracted data, capturing and categorizing major trends in findings. A semantic approach was used, where we looked to the explicit meanings of the data as presented by the authors, rather than impose our own meanings onto the text.

### Appraisal of Evaluative Literature

To assess the factors associated with the success of the ACO model, we conducted a critical appraisal of the methodology of the evaluative literature. Qualitative methods were appraised using the Consolidated criteria for Reporting Qualitative research (COREQ) Framework [18].

Only quantitative studies that employed a quasi-experimental design were considered ‘higher-quality’. Quasi-experimental designs are defined as studies that combine before-and-after data with a comparison group [19]. Quasi-experimental studies were critically appraised using the methodological criteria derived from the statistical and econometric literature (***Table 1***). In addition, the quality and completeness of reporting in these studies was assessed using the Strengthening the Reporting of Observational Studies in Epidemiology (STROBE) statement [20] (Supplementary file 1).

All of the quasi-experimental studies we reviewed employed a difference-in-differences (DD) method. Underpinning the validity of the DD method are two assumptions: exchangeability and conditional exogeneity [21]. Within DD models, the exchangeability assumption suggests that if the comparison group was swapped with the intervention group in the pre-policy period, the results of the study would not change. Experimental designs achieve exchangeability through randomization. DD studies of policy interventions must rely on the structure of the data, statistical tools, and an in-depth understanding of the policy context. Conditional exogeneity requires that the policy change is not driven by pre-policy outcomes, and that there is an absence of unobserved time-varying effects that impact the policy change and the outcome.

**Table 1:** Criteria for Appraisal of Quasi-Experimental Studies.


CRITERIA	DESCRIPTION

**Exchangeability Domain**	

More than one pre-period	To assess whether trends for the outcome in the pre-policy period are parallel, there is more than one pre-period time point [21,22].

Graphical and/or statistical evaluation of parallel trends	The trends are evaluated graphically and/or statistically to determine if they are comparable [21,23].

Weighted/matched regression	Use propensity score matching or weighting to balance intervention and comparison groups on observable baseline characteristics [24].

Comparison of changes in observable characteristics	Assess changes over time in the composition of the intervention and comparison groups [21].

**No Time-Varying Confounding Domain**	

Test whether pre-policy trend predicts policy change	Use statistical tests to determine whether the trend for the outcome in the pre-policy period predicts the policy change [21].

Control for or discuss potential sources of time-varying confounding	Provide a discussion of sources of potential time-varying confounding (e.g., contemporaneous policy changes) and control for them where possible).

Triple-difference model	Employ a difference-in-difference-in-differences model to control for potential time-varying confounding [25].

**Modelling Domain**	

Different functional forms are considered	If the outcome is non-linear, consider alternative functional forms [26].

Standard errors are adjusted for clustering and serial correlation	Adjust standard errors and inferential statistics for correlation between individuals in a practice/group and within individuals over time [27].

Large number of groups (organizations, regions, practices)	Include a large number of groups (e.g., ACOs) to improve the power of inferential statistics [27].

Placebo testing	Test the robustness of estimates by determining whether the statistical models find an effect in places they should not (e.g., outcomes not affected by policy change, time-periods before policy change) [21,28].


### Limitations

It is important to note that our review of the literature was not systematic and thus should not be treated as comprehensive. Our objective was to assess the key documents that evaluated the implementation of the ACO model, to highlight the core features of ACO models that contribute to their positive impacts, and to consider whether Canada is well-positioned to implement ACO-like models. Reviews of ACO models have been completed by other scholars and we have relied on this work to ensure we have captured key studies [16,17]. Additionally, as ACO models began to be implemented across the US in 2012, few studies have evaluated changes in the “Quadruple-Aim” outcomes beyond four years. As such, there remains a need for studies that evaluate the effects of ACOs on outcomes over longer follow-up periods.

## Description of the policy development and evaluation

### Characteristics of the ACO Model

Canada’s provinces and territories have universal publicly-funded, single-payer systems; therefore, we focus the following discussion on publicly-funded Centers for Medicare and Medicaid Services (CMS) ACO models in the US, as this setting is more comparable.

ACOs have expanded considerably in the recent years in the US. As of December 2020, there were 517 publicly-funded ACOs across 50 states, Washington, D.C., and Puerto Rico, up from 404 in 2015 [29]. The implementation of ACOs has been an iterative process, which has resulted in considerable heterogeneity in designs. The major CMS ACO models include: the Medicare Shared Savings Program (MSSP), the Advanced Savings Model (ASM), the ACO Investment Model (AIM), the Pioneer ACO model, and the Next Generation ACO Model (NGACO). The most common CMS ACO model is the MSSP. In 2015, there was a total of 324 (58% of all ACOs in the US) MSSP ACOs that involved physicians, hospitals, and other facilities [16].

The ASM model, a subgroup of the MSSP, was designed to encourage participation by smaller rural healthcare organizations and ran from 2012 to 2015 [30,31]. In this model, the CMS provided 35 ACOs one up-front payment and 24 monthly payments adjusted for the number of historically-assigned beneficiaries to cover start-up and operational expenses [31]. In April 2015, CMS launched the AIM to (i) establish ACOs in areas with few ACOs, and (ii) provide resources to smaller ACOs (defined as those with <10,000 beneficiaries) to sustain their participation in MSSP and transition from one-sided to two-sided financial risk [32]. AIM is a successor to the ASM, providing upfront and monthly payments to ACOs adjusted for preliminarily prospectively-assigned beneficiaries [32]. Over 70% of AIMs have most of their delivery sites in rural areas [32,33].

The Pioneer ACO model ran as a demonstration project between 2012 and 2016. This model targeted hospitals or provider groups with existing health information technology infrastructure; experience providing coordinated, managed, and patient-centered care; as well as at least 15,000 assigned beneficiaries (5,000 for rural ACOs). The CMS selected 32 hospital and provider groups to participate in the Pioneer demonstration project, with nine remaining in the final evaluation of the model in 2016 [34,35]. Building on the Pioneer and MSSP ACO experience, the NGACO model was launched in January 2016 to enable ACOs to take on higher levels of risk to share in greater financial rewards [36,37].

All of these ACO models have similar methods of sponsorship and membership, they all participate in shared savings incentive programs, and they are all required to meet quality performance targets. ACO models can be sponsored by groups of doctors, hospitals, and other healthcare providers, who voluntarily come together to deliver and coordinate care. ACOs have a minimum of 5,000 beneficiaries assigned to them by the CMS. Beneficiaries can be assigned to an ACO if they receive at least one primary care service from a physician participating in an ACO. Beneficiaries are assigned to the ACO that provides the greatest proportion of their primary care services. The distribution of shared savings is conditional on meeting quality performance targets, spanning the following domains: patient and caregiver experience of care, care coordination and safety, preventive healthcare, and chronic disease management. In recent iterations of the ACO model, providers can share both savings and losses (called “two-sided” ACO models). By taking on greater financial risk, providers are entitled to a larger proportion of the savings. The characteristics of the CMS ACO models are summarized in ***Table 2***.

**Table 2:** Major CMS ACO Models in the US.


MODEL TYPE	RISK AND SHARED SAVINGS	NUMBER OF ACOS	NUMBER OF BENEFICIARIES

Medicare Shared Savings Program, MSSP (2012-ongoing) [29]	One-sided: Share savings with the CMS up to a maximum of 50% (if quality performance standards are met). Two-sided: Larger share of savings in exchange for sharing losses with CMS. Maximum 60% (if quality performance standards are met).	One-sided (2020): 325 Two-sided (2020): 192	Total (2020): 11.2 million Mean per ACO (2020): 21,663

Pioneer ACO Program (2012–2016) [34,35]	Originally less financial risk. Not responsible to pay CMS for any losses during contract period.	32 launched (2012) 9 remaining in 2016	Total (2014): 816,362 Mean per ACO (2014): 35,494

Advanced Savings Model, ASM (2012–2015) [30,31]	One-sided: Share savings only with the CMS 50%. Two-sided: Larger share of savings in exchange for sharing loses with CMS. Savings/loss rates: 2–3.9% based on ACO size (difference between an ACOs benchmark and actual spending).	36 launched (2012) 33 remaining in 2015	Total (2014): 288,278 Mean per ACO (2014): 8,237

ACO Investment Model, AIM (2015–2020) [32,33]	Purpose of AIM is to enable smaller/rural ACOs to transition from one-sided to two-sided risk, wherein they become liable for paying CMS a percentage of Medicare spending above their benchmark.	45 launched (2015) 14 remaining in 2020 9 moved to two-sided risk by 2019 and 7 of these remained in 2020	Total (2017): 487,000 Mean per ACO (2017): 10,822

Next Generation ACO, NGACO (2016-ongoing) [36,37]	Providers take on higher levels of financial risk for greater rewards. If spending exceeds benchmark 80–100% loss share rate. If spending is below the benchmark 80–100% savings share rate. Physicians eligible for 5% bonuses starting in 2019.	18 launched (2016) 41 operating in 2019	Total (2019): 1,399,398 Mean per ACO (2019): 34,132


### Characteristics Linked to the Success of Accountable Care Organizations

An overview of the reviewed articles is presented in the Supplementary file 2. Our thematic analysis identified six themes containing factors linked to high-performing ACO models: (i) global budgets, accountable quality contracts, and incentives; (ii) independent physician group- versus hospital-led ACOs; (iii) baseline outcomes; (iv) physician turn over; (v) shifting care to outpatient settings; and (vi) risk. ***Table 3*** summarizes which of the reviewed articles discussed the identified success factors.

**Table 3:** Summary of ACO Success Factors.


STUDY*	GLOBAL BUDGETS, AQC, AND INCENTIVES	INDEPENDENT PHYSICIAN GROUP-LED ACOS	HOSPITAL-LED ACOs	BASELINE OUTCOMES AND STARTING POINTS	CONSISTENCY OF CARE AND PROVIDER BUY-IN	SHIFTING CARE TO OUTPATIENT SETTINGS	RISK**

Barry (2015) [72]						✔	

Borza (2019) [57]			✔	✔		✔	

Chien (2014) [42]	✔						

Christensen (2016a) [45]	✔				✔		

Christensen (2016b) [64]	✔		✖		✔		

Colla (2016) [60]				✔			

Colla (2019) [70]						✔	

Geyer (2016) [54]		✔	✔				

Huskamp (2016) [46]	✔						

Joyce (2017) [73]	✔						

Kelleher (2015) [55]		✔	✔			✔	

Lowell (2018) [71]			✖				✖

McWilliams (2013) [51]	✔						

McWilliams (2014) [61]				✔			

McWilliams (2015) [58]			✖	✔			

McWilliams (2016) [52]		✔	✖				

McWilliams (2017) [74]			✖				

McWilliams (2018) [53]		✔					

Nyweide (2015) [62]				✔	✔	✔	

Resnick (2018) [44]	✔						

Rutledge (2019) [47]	✔				✔		

Ryan (2017) [56]		✔	✔				

Song (2011) [43]	✔			✔		✔	

Song (2012) [59]	✔			✔		✔	✔

Song (2017) [48]		✔					

Stuart (2017) [49]	✔						

Trombley (2019) [63]				✔		✔	


* This table only lists studies that have demonstrated a positive impact of ACOs on at least one pre-specified outcome. ** Since very few ACOs assumed 100% risk in the first year of operation, whether assuming 100% risk was associated with reduced spending remained unclear. However, the few ACOs that did assume 100% risk showed significantly lower Medicare spending. Legend: ✔ = factors linked to success of ACOs, as identified by thematic analysis; ✖ = factors that challenged the success of ACOs, as identified by thematic analysis; blank = factors not discussed in the study. Abbreviations: Alternative Quality Contract, AQC.

### Global Budgets, Accountable Quality Contracts, and Incentives

Studies highlighted that a shift from fee-for-service reimbursement to global budget funding models was a contributing factor to successful ACO outcomes. Global budgets are fixed prospective payments that cover operating expenses for a specific period of time, and are often based on historical budgeting, but can also be adjusted to account for factors like patient case-mix and volume [38]. However, studies also cautioned that if the funding arrangements do not target specific care processes and outcomes to clinically relevant indicators, both quality and cost management may not improve [39,40,41,42,43,44]. Studies spoke to the importance of clinically relevant quality indicators that focus on both outcomes and processes in order to improve quality, reduce “stinting of care” (i.e., providing limited care to achieve spending targets), and support disadvantaged populations [43,44,45,46,47,48,49].

Compared to fee-for-service programs, global budgets were thought to “unlock” the benefits of ACOs [50]. Huskamp et al., (2016) suggested that five-year global budgets provide physicians with the flexibility to coordinate care in a way that traditional fee-for-service models cannot [46]. McWilliams et al., (2013) suggested that these payment schemes increased provider engagement in activities like changing referral processes, focusing on high-risk case management, and redesigning care patterns to reduce waste [50].

### Independent Physician Group- versus Hospital-Led Accountable Care Organizations

There remains disagreement in the literature about whether it is optimal for ACOs to be led by independent physician groups or by hospitals. ACO models led by independent physician groups were linked to high performance in terms of cost savings [48,52,53], while hospital-led ACOs were not [54,55,56]. This was attributed to stronger incentives for physician group practices to lower inpatient and hospital outpatient spending [52,53]. Studies also highlighted the value of having larger organizations (e.g., hospitals) lead integration efforts [54,55,56]. These studies focused on the capacity of ACOs to navigate broader health system structures rather than their ability to achieve savings. Individual physician groups, patient-centered medical homes, and insurers were seen to be limited in their ability to independently track healthcare use across systems [55]. We identified three factors that these studies highlighted as being associated with the success of hospital-led ACOs; information tracking and sharing [54,55,56], guideline and procedure usage across a larger community of actors [55,57], and integration of networks of physicians [55].

### Baseline Outcomes and Starting Points

ACOs with different baseline outcomes (or starting points) experienced different degrees of cost-savings. In particular, some ACOs that targeted high-cost patients would not always achieve sustainable outcomes beyond the first few years of implementation [58,59]. Studies stressed that much of the cost-savings found in the first two years were attributed to an initial focus on case management for high-cost, high-risk, clinically vulnerable, and medically complex patients [43,57,60,61,62,63].

### Consistency of Care and Provider Buy-in

High turnover rates of physicians participating in ACOs were linked to fewer cost-savings over the first two years of the model [62]. On the other hand, receiving consistent primary care from an ACO for over 12 months was associated with reduced costs, inpatient days, and readmission rates to the discharging hospitals, though, due to short study follow-up periods, it remains unclear whether these changes persisted beyond 24 months. Moreover, longer patient attribution to an ACO was associated with increases in outpatient utilization and prescribing [64].

There was agreement within the literature that involvement of non-physician staff is an ACO success factor, and that meaningful engagement and formalized interdisciplinary teams were necessary components for accomplishing this goal [41,47,65,66,67,68,69]. Indeed, expanding teams to include non-physician staff and developing a formalized approach to broaden scopes of practice was thought to offset work-load and administrative burden related to changes in care approaches and reporting requirements, which, in turn, may reduce employee turnover [39].

### Shifting Care to Outpatient Settings

A decline in inpatient utilization among ACO-aligned beneficiaries was linked to spending reductions [55,57,62,63]. This decline in inpatient utilization was suggested to be owed to referrals to lower-cost outpatient settings [62,70].

### Risk

Two studies suggested that ACOs that assumed higher financial risk for over-spending were able to reduce spending at a greater level than those that took on less risk [59,71]. However, the authors noted that this relationship may be questionable since very few ACOs assumed high amounts of risk in their first year of operation, which limited the time-frame for evaluation.

### Characteristics of ACOs Supported by High Quality Evaluative Studies

To assess whether higher quality evidence supports the identified ACO success factors, we performed a critical appraisal of the methodology of the reviewed studies. Based on this appraisal, we highlighted the findings of seven high quality studies (***Table 4***).

**Table 4:** Findings from Seven Higher Quality Quasi-Experimental Studies.


STUDY	SUMMARY OF FINDINGS

Song (2011) [43]	The implementation of the Blue Cross Blue Shield of Massachusetts AQC was associated with “modest slowing of spending growth and improved quality.” While a higher-quality study based on our criteria, the authors only observed one year of outcomes post-implementation.

McWilliams (2013) [51]	Studied the impact of the AQC observing two years of implementation (2009 and 2010) and two years post-implementation. The authors found that the implementation of the AQC was associated with lower spending after the second year, particularly in outpatient care, procedures, imaging, and tests. They also found associations with improvements in some quality of process measures for diabetes and cardiovascular disease, but not with hospitalization, readmission, or cancer screening.

McWilliams (2016) [52]	Evaluated the performance of MSSP ACOs and compared primary care groups to hospital-integrated groups. The authors found that the introduction of the MSSP ACOs was associated with reduced Medicare spending by the ACOs that entered the MSSP in 2012, but not those that entered in 2013. Generally, savings were greater among primary care groups than hospital-integrated groups. The authors found mixed results on measures of quality.

McWilliams (2017) [74]	This study evaluated the impact of the MSSP on post-acute care spending and utilization. The authors found that participation in an MSSP was associated with reductions in post-acute care spending without any reduction in care quality.

Song (2017) [48]	Studied the impact of the AQC on spending and quality of process and outcome measures comparing enrollees with both lower- and higher socioeconomic statuses. The difference-in-difference-in-differences approach was used to compare enrollees to non-enrollees across these socioeconomic strata. Their findings suggested that the implementation of the AQC was generally associated with improvements in quality of process measures, and that the magnitude of the improvement was higher among those of lower socioeconomic status. However, the authors found no difference in outcome measures or spending across SES strata.

McWilliams (2018) [53]	This study evaluated the impact of the MSSP after three years of operation. In particular, the authors studied whether the savings achieved by early adopters were replicated by newer ACOs. The authors found that participation in the MSSP was associated with reductions in Medicare spending among physician-led groups, but not among hospital-integrated ACOs.

Resnick (2018) [44]	This study evaluated the impact of MSSP ACO enrollment on changes in appropriate cancer screening rates. Appropriateness was determined based on patient age and predicted survival. If screening increased for those who would most benefit and decreased for those who would not, then appropriateness was improved. The authors found that enrollment in an MSSP ACO was associated with “modest” improvements in appropriate breast and colorectal cancer screening. MSSP ACO enrollment was also associated with decreased prostate cancer screening regardless of age or predicted survival.


Abbreviations: Alternative Quality Contract, AQC; Medicare Shared Savings Program, MSSP; Socioeconomic status, SES.

Several of these studies focused on the Accountable Quality Contract (AQC) model [43,51]. The AQC is a private contracting model for ACOs developed by Blue Cross Blue Shield of Massachusetts (BCBSMA) in 2009 that served as a blueprint for subsequent ACO models rolled out nation-wide. The AQC is primarily physician-led, uses global payments mixed with pay for performance, and is similar to the two-sided CMS models noted previously [17,59]. At the time of writing over 80% of Massachusetts physicians and hospitals participate in AQC [75]. One study of 11 provider organizations that entered the AQC between 2009–2010 demonstrated that the model achieved cost savings, but had mixed results with respect to chronic disease management quality indicators [51]. Studies by Song et al., [2017, 2011] covering 7–17 practices that entered AQC between 2009–2012, examined a broader set of indicators and found that the implementation of the AQC was associated with improvements in some quality measures, but not in patient outcomes (e.g., hospitalization and readmissions) [43,48].

Studies of the MSSP ACOs found evidence of at least modest savings that were maintained over time [52,53,74]. However, these savings seemed to be sustained only for physician-led groups, not hospital-integrated ACOs [52,53]. Evidence regarding improvements in quality of process measures were found, but evidence for improvements in overall quality and patient outcomes was mixed [44,52]. Finally, ACOs that focused on higher-risk populations than those with lower socioeconomic status tended to achieve greater improvements in savings and quality of process measures than ACOs that focused on lower-risk and higher socioeconomic status populations [48].

Overall, based on the thematic analysis, quality appraisal of the evaluative evidence, and the frequency of mention across studies, we isolated three factors that appear to be conducive to optimal ACO performance:

1. There is evidence supporting the potential for improved quality of care and reduced costs with physician group-led ACOs than with other models. For hospital-led ACOs, financial integration with physicians has been linked to increased chances of cost-savings and quality improvement.
2. Global budgets and Accountable Quality Contracts encourage the development of processes that include estimating the risk of readmission, discharging patients with follow-up, and the use of electronic tools (e.g., electronic medication reconciliation). This payment approach allows for flexibility, and encourages the use of preventive strategies.
3. A focus on cross-sector collaboration and team-based approaches were associated with improved medication reconciliation, reduced service utilization, provider buy-in, meeting the needs of the most vulnerable populations in rural locations, and reduced workplace stress. Additionally, deliberate inclusion of interdisciplinary teams was valued by providers and encouraged their participation in ACO models.

## The US ACO experience and the Canadian Context

There has been vast research describing the process of comparative policy analysis, as well as the interpretation of such analysis and implications for policy implementation [15,76,77,78,79,80]. In line with much of Marmor’s (2005, 2017) work, the present research relies heavily on offering a descriptive assessment of the experiences of ACOs in the US and the current context in Canada to not only simplify “muddled language”, but also to discuss contextual differences that may impact the applicability of the ACO experience in Canada [15,76]. To do this, in the previous section of the paper, we identified and described key features of ACO models and highlighted elements of effective models based on the reviewed literature.

Canadian governments are interested in the ACO model, in essence, to help achieve the goals of the “Quadruple-Aim”. Relying on the findings of the thematic analysis that identified key factors of success, in this section, we compare the core factors with the current Canadian context to gain insight into their possible applicability and transferability within Canada.

In Canada, the federal government shares responsibility for the funding of healthcare services, while each of the thirteen provinces and territories manage their own universal single-payer healthcare systems. Hospital and physician services hold a privileged position within Canadian Medicare. Under the *Canada Health Act* (1985), provinces are required to cover their beneficiaries for the full cost of medically necessary hospital and physician services in order to be eligible for federal cash transfers. Provinces may also cover other healthcare services, such as long-term care and mental health and addictions care (not provided in hospitals or by physicians), in their provincial health insurance programs, though coverage varies across the country. This means that the majority of hospital and physician services are publicly funded (including inpatient and physician-delivered mental health services). Yet, hospitals are independent not-for-profit organizations (in Ontario, Canada’s most populous province), and physicians generally work in independent practices (including a minority working in group practices) and are paid mostly on a fee-for-service basis with no mechanism to hold physicians accountable for quality or efficiency-related outcomes [81]. This system of public funding and private delivery has contributed to fragmentation of service delivery, both within and across sectors [82].

### Insight 1: Physician-led Accountable Care Organizations

The longstanding independence of physicians in Canada poses some challenges for the implementation of a physician group-led ACO model. In Canada, physician services are largely disconnected from other health and social services, which can make it challenging for physician groups to adopt a leadership role in coordinating services for populations and/or in securing the participation of community-based physician practices [83].

The implementation of an ACO-like model would not be the first time provincial/territorial governments have attempted to improve coordination of care across hospital and community-based sectors. In Alberta, the Primary Health Care Integration Network connects zones of Alberta Health Services — the central planning body for the delivery of healthcare services in Alberta — with primary care teams (called Primary Care Networks or PCNs), Strategic Clinical Networks, Alberta Health (a government department), and academic partners [84]. This initiative is supported by an amended (2016) master agreement between the Alberta Medical Association, the Government of Alberta, and the Alberta Health Services [85]. The ultimate goal of PCNs is to achieve improvements in the “Triple–Aim” objectives by enhancing transitions between care settings [86]. While this did not necessarily overcome the challenges related to physician independence or produce transformative change in terms of integrating the full continuum of care in Alberta, PCNs have successfully created a culture of shared responsibility between providers and payers, which is unique in Canada [87,88,89].

In 2012, the Government of Ontario implemented the Health Links initiative. This is a decentralized planning initiative, organized by volunteering health and social care organizations [90]. Health Links aimed to improve coordination and reduce duplication of care by connecting health and social care providers to develop shared care plans for enrolled patients. Unfortunately, Health Links experienced difficulty securing the participation of solo and group physician practices who lacked capacity to engage in the coordination activities of the Health Links program [91]. In 2019, Ontario introduced Ontario Health Teams (OHTs) that are being phased into operation over the course of 2020/2021.

The degree to which the aforementioned models will achieve physician integration and leadership remains unknown. The difficulty experienced with the Ontario initiatives likely lies in the “founding bargain” between the medical profession and the government. In Ontario, where community-based physicians mostly work in independently owned and operated practices, efforts to achieve such integration may be complicated by the fragmentation of the medical sector, which privileges physician independence, and could challenge reforms to funding arrangements without direct engagement with the medical profession [88].

While this approach was not favored in the literature, Canadian governments could lean on hospital-led team-based practices to take charge in regional planning initiatives, since the majority of hospitals (outside of Ontario) do not maintain the degree of independence that physicians do [92]. Given the nature of physician independence in the Canadian context, such an approach may be the most feasible.

### Insight 2: Global Budgets and Alternative Payment Models

The literature on ACOs suggests the need to move away from fee-for-service funding to a global budget model that is integrated across providers and sectors. For most jurisdictions in Canada, this would require significant payment reform for physician services. In 2017, 73% of clinical payments to physicians were fee-for-service payments, ranging from 3% in the Northwest Territories to 87% in Alberta. The proportion of physicians whose payment consisted of more than 50% fee-for-service payment was 74% in the reporting jurisdictions [93].

There is some precedent for significant payment reform for physicians in Canada. Ontario implemented a series of alternative payment models between 1999 and 2012 that resulted in the majority of primary care physicians opting out of traditional fee-for-service payment. This demonstrates the possibility and opportunity for future reform efforts and for other provinces to implement reform strategies similar to Ontario. However, fee-for-service remains the dominant method of paying specialist physicians [88].

The majority of Alberta’s physicians remain on fee-for-service payment schemes. However, with the development of PCNs, there have been attempts to achieve coordination through aligning incentives among providers and payers, which is a unique approach for Canada and demonstrates potential for shared accountability among payers and providers [87,89,94,95].

### Insight 3: Team-based Approaches

Given the evidence supporting physician-led ACOs and the formal inclusion of interdisciplinary teams, Canadian provinces have laid the groundwork for further reform in this direction. However, as noted in insights 1 and 2, the spread of primary care to reach beyond its silo into other community health spaces has been less effective [96,97,98]. Notably, Ontario – the province that has made arguably the most progress toward implementing team-based primary care reforms – could build on its existing team-based care (e.g., family health teams and community health centers) approaches. Unfortunately, the majority of these approaches do not formally partner with social care or community support organizations, which was a key factor for demonstrating cost-reductions in the US literature (e.g., receiving care in outpatient settings). Though these types of primary care transformations have been seen across Canada, they remain outliers, and they require voluntary engagement and strong government and professional leadership working in symphony. In order to make these approaches bridge the health and social care divide, there would need to be inclusion of and buy-in from community-based and social care providers and strong transdisciplinary collaboration [99,100,101].

## Conclusions

Canadian jurisdictions are considering the implementation of ACO-like models, with Ontario Health Teams being the most recent example. OHTs, on paper, seem to mimic the partnerships achieved though the Integrated Care Systems (ICS) in England’s NHS [102]. This review of the literature on the ACO experience in the US has revealed a limited set of lessons for Canadian decision-makers. If Canadian jurisdictions were to move ahead with an ACO-like model, the literature suggests that they should be physician-led, that they should involve a move away from fee-for-service payment, and that they should formalize team-based approaches to care. Our review also suggests that although such reforms are not unprecedented in the Canadian context, they would require significant reform to the status quo. Specifically, these efforts would need to reform the majority of physician payment models, formally encourage health and social care collaboration, and build shared accountability opportunities (either through payment models or clinically relevant performance measurement assessments).

On the other hand, this limited set of lessons suggests that there is an overall lack of clarity around what makes the ACO model successful. Building off existing reviews [103], future research should examine whether the implementation of ACO-like models in other jurisdictions, such as ICS in England’s NHS, has produced favorable outcomes, to infer lessons for Canada’s adoption. While there are many concerns that mostly focus on public/private mixes, ACOs offer insight in a variety of approaches that could encourage increased focus on population health, as well as a shift away from episodic reactive care towards chronic disease management and preventive care.

## Additional Files

The additional files for this article can be found as follows:

10.5334/ijic.5677.s1 Supplementary file 1. Quality Assessment of Seven High Quality Quasi-Experimental Studies.

10.5334/ijic.5677.s2 Supplementary file 2. Characteristics of the included evaluative studies.
