# Supplementary material for: What Can Canada Learn From Accountable Care Organizations: A Comparative Policy Analysis
Source: Int J Integr Care. 2022 Apr 1;22(2):1. doi: 10.5334/ijic.5677 (PMC8992768; doi:10.5334/ijic.5677)
Supplement: Supplementary file 1. — Quality Assessment of Seven High Quality Quasi-Experimental Studies. [file ijic-22-2-5677-s1.pdf]

Supplementary 1. Quality Assessment of Seven High Quality Quasi-Experimental Studies.

Study	Summary of Findings	Sample size (n)	Comparison	Quality Assessment
Song 2011	The implementation of the Blue Cross Blue Shield of Massachusetts AQC was associated with "modest slowing of spending growth and improved quality." While a higher-quality study based on our criteria, the authors only observed one year of outcomes post-implementation.	Intervention group (n = 380,142)<br><br>Comparison group (n = 1,351,446)	Intervention: Blue Cross Blue Shield enrollees with PCP in AQC in 2009<br><br>Comparison: Blue Cross Blue Shield enrollees with PCP not the AQC	STROBE reporting; >1 pre-policy time period; Evaluation of parallel trends; propensity score weights; non-linear functional forms tested; cluster adjusted standard errors; Comparison of changes in observable characteristics
McWilliams 2013	Studied the impact of the AQC observing two years of implementation (2009 and 2010) and two years post-implementation. The authors found that the implementation of the AQC was associated with lower spending after the second year, particularly in outpatient care, procedures, imaging, and tests. They also found associations with improvements in some quality of process measures for diabetes and cardiovascular disease, but not with hospitalization, readmission, or cancer screening.	n = 1,761,325 person-years	Intervention: beneficiaries with a plurality of visits in seven provider organizations in the AQC in 2009 and four in the AQC in 2010<br><br>Comparison: beneficiaries with a plurality of visits with nonparticipating organizations	STROBE reporting; >1 pre-policy time period; evaluation of parallel trends; propensity score weights; non-linear functional forms tested; cluster adjusted standard errors; control/discuss time-varying confounding; comparison of changes in observable characteristics
McWilliams 2016	Evaluated the performance of MSSP ACOs and compared primary care groups to hospital-integrated groups. The authors found that the introduction of the MSSP ACOs was associated with reduced Medicare spending by the ACOs that entered the MSSP in 2012, but not those that entered in 2013. Generally, savings were greater among primary care groups than hospital-integrated groups. The authors found mixed results on measures of quality.	n = 15,808,286 person years	Intervention: beneficiaries with plurality of visits with 220 ACOs entering the MSSP in 2012 or 2013<br><br>Comparison: beneficiaries with a plurality of visits with non-ACO providers	STROBE reporting; >1 pre-policy time period; evaluation of parallel trends; propensity score weights; non-linear functional forms tested; cluster adjusted standard errors; control/discuss time-varying confounding; comparison of changes in observable characteristics
McWilliams 2017	This study evaluated the impact of the MSSP on post-acute care spending and utilization. The authors found that participation in an MSSP was associated with reductions in post-acute care spending without any reduction in care quality.	25,544,650 (19% on average in ACOs)	Intervention: beneficiaries with a plurality of visits with providers in MSSP ACOs (separately examined cohorts of ACOs entering MSSP in 2012, 2013 and 2014).<br><br>Comparison: beneficiaries in the same service areas with plurality of visits with nonparticipating	STROBE reporting; >1 pre-policy time period; evaluation of parallel trends; propensity score weights; cluster adjusted standard errors; large number of groups; control/discuss time-varying confounding; comparison of changes in observable characteristics

			providers	
Song 2017	Studied the impact of the AQC on spending and quality of process and outcome measures comparing enrollees with both lower- and higher socioeconomic statuses. The Difference-in-difference-in-difference approach was used to compare enrollees to non-enrollees across these socioeconomic strata. Their findings suggested that the implementation of the AQC was generally associated with improvements in quality of process measures, and that the magnitude of the improvement was higher among those of lower socioeconomic status. However, the authors found no difference in outcome measures or spending across SES strata.	ACO<br>low SES<br>(n=299,285)<br>high SES<br>(n=244,415)<br><br>Non-ACO<br>low SES<br>(n=1,053,089)<br>high SES<br>(n=650,041)	Comparison: lower-socioeconomic status (assigned via principal components analysis) Blue Cross Blue Shield enrollees in ACQ in 2009 vs. higher-socioeconomic status Blue Cross Blue Shield enrollees in ACQ in 2009.<br><br>Triple difference model included comparison to non-AQC Blue Cross Blue Shield enrollees.	>1 pre-policy time period; evaluation of parallel trends; cluster adjusted standard errors; triple difference model
McWilliams 2018	This study evaluated the impact of the MSSP after three years of operation. In particular, the authors studied whether the savings achieved by early adopters were replicated by newer ACOs. The authors found that participation in the MSSP was associated with reductions in Medicare spending among physician-led groups, but not among hospital-integrated ACOs.	n = 29,616,964 person years (19.6% on average in ACOs)	Intervention: beneficiaries who had a plurality of visits with a primary care physician in an ACO<br><br>Comparison: beneficiaries who had a plurality of visits with a provider not in an ACO	STROBE reporting; >1 pre-policy time period; evaluation of parallel trends; propensity score weights; cluster adjusted standard errors; control/discuss time-varying confounding; comparison of changes in observable characteristics; placebo testing
Resnick 2018	This study evaluated the impact of MSSP ACO enrollment on changes in appropriate cancer screening rates. Appropriateness was determined based on patient age and predicted survival. If screening increased for those who would most benefit and decreased for those who would not, then appropriateness was improved. The authors found that enrollment in an MSSP ACO was associated with "modest" improvements in appropriate breast and colorectal cancer screening. MSSP ACO enrollment was also associated with decreased prostate cancer screening regardless of age or predicted survival.	n = 56,470,997 person years (13,460,798 in average in ACOs)<br><br># ACOs not reported	Intervention: beneficiaries who had a plurality of visits with a primary care physician in an ACO<br><br>Comparison: beneficiaries who had a plurality of visits with a provider not in an ACO	STROBE reporting; >1 pre-policy time period; cluster adjusted standard errors; control/discuss time-varying confounding; comparison of changes in observable characteristics; triple-difference model

Abbreviations: Alternative Quality Contract, AQC; Medicare Shared Savings Program, MSSP; Socioeconomic status, SES
