# Supplementary material for: What Can Canada Learn From Accountable Care Organizations: A Comparative Policy Analysis
Source: Int J Integr Care. 2022 Apr 1;22(2):1. doi: 10.5334/ijic.5677 (PMC8992768; doi:10.5334/ijic.5677)
Supplement: Supplementary file 2. — Characteristics of the included evaluative studies. [file ijic-22-2-5677-s2.pdf]

Supplementary file 2. Characteristics of the included evaluative studies.

Study	Model	Comparison group?	Design	Study period	Population	Summary of main findings
ASCP 2014[1]	ACO, NS	No	Qualitative	2014	Clinical pharmacists	Two key areas where pharmacists have an opportunity to get involved are medication reconciliation and transition of care. Other areas may include patient education and reducing readmission rates. Adequate integration of pharmacists in ACOs will involve focusing on team-based care within ACOs and informing physicians of the pharmacy scope of practice.
Bagwell 2017[2]	ACO, RHC	No	Qualitative	2012-2013	ACO Administrators	Predominant themes: 1) ACOs are growing in size and number and have various organizational structures; 2) there is an expanding emphasis on preventive primary care and chronic disease management for patients; and 3) there is a need for improved information technology integration with clinical services and financial systems.
Barry 2015[3]	MSSP, Pioneer	Yes	Quasi-experimental Mixed methods	2006-2011	Adult beneficiaries	Reduced spending on mental health hospital admissions. No changes in total mental health spending.<br><br>No changes in hospital readmissions, outpatient follow-up after a mental health admission, rates of depression diagnosis, number of enrollees with a mental health diagnosis.
Borza 2019[4]	MSSP	Yes	Quasi-experimental	2010-2014	Adult beneficiaries	Significant reductions in 30-day risk-adjusted hospital readmissions among surgical patients in ACO-affiliated hospitals, compared to non-ACO hospitals.
Busch 2016[5]	MSSP, Pioneer	Yes	Quasi-experimental	2008-2013	Beneficiaries with mental health dx	Reduced spending on mental health hospital admissions. No changes in total mental health spending. No changes in hospital readmissions, outpatient follow-up after a mental health admission, rates of depression diagnosis, number of enrollees with a mental health diagnosis.
Castle 2016[6]	ACU	No	Descriptive	2009	None	The ACU is a "disruptive innovation" that radically reconfigures the way care is delivered on inpatient acute care units. The model dissolves the barriers to interprofessional collaborative, team-based, patient-centered care that confront nurses, physicians, and other health care providers on traditional units.
Chien 2014[7]	AQC	Yes	Quasi-experimental	2006-2010	Pediatric beneficiaries	Increase in pediatric care quality composite measure in AQC, relative to non-AQC patients during the first 2 years of the contract.
Christensen 2016a[8]	Pediatric Medicaid ACO	No	Retrospective cohort	2013-2015	Pediatric beneficiaries	Continuous receipt of pediatric primary care services from ACO for 2 years associated with reduced inpatient days, but increase in office

						visits, emergency department visits, and use of pharmaceuticals. Changes in health service utilization were mirrored by cost reductions.
Christensen 2016b[9]	Pediatric Medicaid ACO	No	Retrospective cohort	2013-2015	Pediatric beneficiaries	Continuous receipt of pediatric primary care services from ACO for 2 years associated with reduced 30-day readmission rate at hospitals other than the discharging hospital, but not the discharging hospital. Reduction in the cost per inpatient episode of care for all patients.
Chukmaitov 2018[10]	MSSP, Pioneer	Yes	Quasi-experimental	2010-2013	Hospitals	A decrease was found in preventable hospitalizations for COPD and asthma and for diabetes complications for ACO participating hospitals, but no significant differences for preventable CHF hospitalizations and 30-day readmissions.
Colla 2016[11]	MSSP	Yes	Quasi-experimental	2009-2013	Adult beneficiaries	Reduction in beneficiary-level spending in ACO Medicare beneficiaries and clinically vulnerable subgroup. Reduction in hospitalizations, emergency department visits in both general and clinically-vulnerable ACO groups.
Colla 2019[12]	ACO, NS	Yes	Quasi-experimental	2009-2014	Adult beneficiaries	Reductions in post-acute discretionary care costs in beneficiaries attributed to ACO vs. non-ACO providers. Discretionary care was defined as admission to skilled nursing facilities or receipt of inpatient rehabilitation among pneumonia patients, in whom post-acute inpatient rehabilitation is not the standard of care.<br><br>Reduced length of stay in skilled nursing facilities among hip fracture, stroke, and pneumonia patients after ACO implementation.
Comfort 2018[13]	MSSP	No	Descriptive	2017	ACO practices	There is greater heterogeneity within ACO types than between ACO types. There were no consistent differences on core quality indicators by ACO type, nor were there differences in likelihood of achieving savings or overall spending per-person-year. There was evidence for higher spending on physician services for physician-led ACOs.
D'Aunno 2018[14]	MSSP	No	Mixed methods	2011-2012	ACO leadership	Factors that distinguish high- from low-performing ACOs: (1) collaboration with hospitals; (2) effective physician group practice prior to ACO engagement; (3) trusted, long-standing physician leaders focused on improving performance; (4) sophisticated use of information systems; (5) effective feed-back to physicians; and (6) embedded care coordinators.
Dupree 2014[15]	MSSP, Pioneer	No	Mixed methods	2012	ACO leadership	ACOs have so far devoted little attention to surgical care. Instead, they have emphasized coordinating care for patients with chronic conditions and reducing unnecessary hospital readmissions and ED visits. In the years to come,

						ACOs will likely focus more on surgical care. Some ACOs are able to affect surgical practice patterns through referral pressures, but local market conditions may limit ACOs' abilities to alter surgeons' behavior.
GAO 2015[16]	Pioneer	No	Descriptive	2012-2013	Adult beneficiaries	Over half of ACOs earned savings during both periods. Each amount is 4% of total expenditures for the ACOs that produced shared savings in each year.<br><br>ACOs that participated in both years had higher quality scores in 2nd year for 22/33 quality measures.
Geyer 2016[17]	Pioneer	Yes	Retrospective cohort	2005-2009	Adult trauma patients	Reduced cost of hospitalization.<br><br>Reduced number of imaging studies during hospitalization.<br><br>No difference in mortality, ICU length of stay, overall hospitalization length of stay.
Gittel 2015[18]	ACO, NS	No	Literature review	2013	Studies on teamwork	Health care systems and change agents seeking to respond to the challenges of accountable care can use TeamSTEPPS as a validated multilevel teamwork intervention methodology, enhanced by relational coordination as a validated multilevel teamwork measure with diagnostic capacity to pinpoint opportunities for improving teamwork along specific dimensions (e.g., shared knowledge, timely communication) and in specific role relationships (e.g., nurse/medical assistant, emergency unit/medical unit, primary care/specialty care).
Green 2015[19]	Pioneer	Yes	Quasi-experimental	2012-2013	Adult beneficiaries	10 ACOs showed savings in both performance years evaluated.
Green 2016[20]	ASM	Yes	Quasi-experimental Mixed methods	2012-2014	Adult beneficiaries	No significant change in quality indicators or Medicare spending. More AP ACO physicians were involved in decision to participate in ACO, agreed that the ACO model is effective at providing high-quality cost-effective care, and that remaining competitive in the local market is important than physicians in other ACO.
Herrell 2016[21]	MSSP	Yes	Quasi-experimental Mixed methods	2011-2013	Older adult beneficiaries, cancer patients	No difference in 30-day mortality rate, complication rate, prolonged length of stay rate, and adverse perioperative outcomes.
Hewner 2016[22]	Medicaid ACO	Yes	Retrospective cohort	2012	Adult beneficiaries	Reduced 90-day rehospitalizations (inpatient and emergency department treat-and-release).<br><br>Initial hospitalization in a ACO-affiliated hospital was associated with longer time to readmission in the first 90 days post-discharge.
Hofler	ACO, RHC	Yes	Descriptive	2012-	RHCs	Joining an ACO raises cost/visit. The jump in

2016[23]				2013		cost can be substantial, with point estimates ranging from 14% to nearly 21%. This increase lasts at least two years. The rise seems to be less in the second year.
Hsu 2017[24]	Pioneer	No	Descriptive	2012-2014	Physicians and beneficiaries	Few beneficiaries per physician in ACOs (<5% of a typical panel). Substantial physician turnover, with more than half either joining or leaving the contract during the study period. When physicians left the ACO, most of their attributed beneficiaries also left the ACO. Conversely, about half of the growth in the beneficiary population was because of new physicians affiliating with the ACO; the remainder joined after switching physicians.
Huskamp 2016[25]	AQC	Yes	Quasi-experimental	2006-2011	Adult beneficiaries	Increased rates of tobacco cessation treatment use overall, among enrollees at risk for tobacco-related complications, and among behavioural health service users.
Joseph 2017[26]	ACO, NS	No	Literature review	2009-2016	Studies on pharmacists	Barriers to integrating pharmacists in primary care ACOs: lack of awareness of pharmacist roles in primary care; complex laws and regulations surrounding clinical protocols, such as collaborative practice agreements; provider status that allows compensation for pharmacist services; and limited access to medical records. By understanding and maximizing the role of pharmacists, several opportunities exist to better manage the medication-use process in value-based care settings.
Joyce 2017[27]	AQC	No	Quasi-experimental	2006-2011	Pediatric beneficiaries with ADHD	Increase in outpatient visits, number of medication management visits among children and youths with ADHD in the AQC group.<br>No difference in spending and no evidence of reductions in health service utilization.
Kelleher 2015[28]	Pediatric Medicaid ACO	Yes	Quasi-experimental	2008-2013	Pediatric beneficiaries	Reduced growth in costs over time per beneficiary. Increase in quality of care on 5 quality measures (including 2 composite measures), decline on 3 quality measures, and no difference on remaining 8 quality indicators.
Kleiner 2017[29]	MSSP	No	Retrospective cohort	2009	Physicians	While most physician practices would fall below the threshold that could raise anticompetitive concerns, this varies considerably by market and specialty. Furthermore, the largest physician practice in most markets potentially remains at risk for antitrust review under the existing criteria
Lowell 2018[30]	NGACO	Yes	Retrospective cohort	2013-2017	Adult beneficiaries	Decrease in hospital length of stay and number of outpatient and wellness visits. Reduction in Medicare spending (reduction in post-acute care spending was the most important contributor). Value of preventive care, comprehensive care

						management, and provider partnerships emphasized in interviews.
McConnell 2017[31]	Oregon's CCO	Yes	Quasi-experimental	2010-2014	Adult beneficiaries	Oregon: reduction in emergency department visits, primary care visits, and acute preventable hospital admissions. Improvement in 3 of 4 measures of access and 1 of 4 measures of appropriateness of care.<br>Oregon's and Colorado's Medicaid ACO models exhibited similar performance on standardized expenditures for selected services. Oregon's model, marked by a large federal investment and movement to global budgets, was associated with improvements in some measures of utilization, access, and quality.
McWilliams 2013[32]	AQC	Yes	Quasi-experimental	2007-2010	Adult beneficiaries	Lower spending per beneficiary following exposure to the AQC. This was largely driven by lower spending on outpatient care in year 2, particularly for beneficiaries with 5 or more conditions. Annual rates of low-density lipoprotein cholesterol testing among beneficiaries with diabetes and cardiovascular disease improved; no sig. changes on other quality metrics.
McWilliams 2014[33]	MSSP, ASM	Yes	Quasi-experimental	2009-2013	Adult beneficiaries	After ACO contracts began, patients' reports of timely access to care and their primary physicians' being informed about specialty care differentially improved in the ACO group. Patients' ratings of physicians, interactions with physicians, and overall care did not differentially change. Among patients with multiple chronic conditions and high predicted Medicare spending, overall ratings of care differentially improved in the ACO group as compared with the control group.
McWilliams 2016[34]	MSSP	Yes	Quasi-experimental	2009-2013	Adult beneficiaries	Increased total and per beneficiary savings; savings consistently greater in independent primary care groups than in hospital-integrated groups among 2012 and 2013 MSSP entrants. No difference on the use of low-value services. But Improvement on some quality indicators.
McWilliams 2017[35]	MSSP	Yes	Descriptive	2009-2014	Adult beneficiaries	Reduction in post-acute spending driven by reductions in acute inpatient care, discharges to facilities other than home, and length of skilled nursing facility (SNF) stays. No difference in 30-day readmissions, use of highly rated SNF, or mortality.
McWilliams 2018a[36]	AIM	No	Descriptive	2015-2016	Adult beneficiaries	Reduction in beneficiary-level and aggregate Medicare spending. Reduction in spending mirrored by reduced acute care inpatient visits, reduced post-acute care days, reduced emergency visits.
McWilliams	MSSP	Yes	Quasi-	2009-	Adult	After 3 years of the MSSP, participation in

2018b[37]			experimental	2015	beneficiaries	shared-savings contracts by physician groups was associated with savings for Medicare that grew over the study period, whereas hospital-integrated ACOs did not produce savings (on average) during the same period.
Muhlestein 2016[38]	ACO, NS	Yes	Descriptive	2013-2016	Hospitals	ACO hospitals performed better on the Hospital Readmissions Reduction Program (HRRP), but not on the Hospital Value-Based Purchasing Program (HVBP) or the Hospital-Acquired Conditions (HAC) Reduction Program. Hospitals joining ACOs did increasingly better than their peers for HRRP but had inconsistent results year-over-year with the HVBP.
Narayan 2016[39]	MSSP	No	Retrospective cohort	2012-2014	Women aged 40-69 years	Increase in biennial mammography utilization (ACO-20 quality indicator), but no correlation with overall ACO-composite quality score. No difference in mammography utilization between ACOs that showed cost savings and those that did not.
Neprash 2017[40]	MSSP, Pioneer	No	Descriptive	2008-2014	Physicians	While the number of hospital mergers and the size of specialty-oriented physician groups increased after the Affordable Care Act was passed, there was minimal evidence that consolidation was associated with ACO penetration at the market level or with physicians 'participation in ACOs within markets. We conclude that payment reform has been associated with little acceleration in consolidation in addition to trends already under way, but there is evidence of potential defensive consolidation in response to new payment models.
Noble 2014[41]	ACO, NS	Yes	Qualitative	2013	ACO administrators	Interviewees working for ACOs most often viewed 'population health' as referring to a defined group of their organisation's patients, though a few applied the phrase to people living in a geographical area. In contrast, interviewees working for public health agencies were more likely to consider 'population health' from a geographical perspective.
Nyweide 2015[42]	Pioneer	Yes	Quasi-experimental	2010-2013	Adult beneficiaries	Reduced utilization of physician services, emergency department, and post-acute care. Lower rate of spending increase (per beneficiary and aggregate). Higher mean score for timely care and clinician communication.
Ortiz 2015[43]	ACO, RHC	No	Descriptive	2007-2011	RHCs	Several characteristics about RHCs indicate that they may be slow to participate in ACOs. However, other characteristics, including their perception that ACOs may improve the quality of care and health outcomes of their patients and communities, may facilitate the process of RHCs joining ACOs, should they choose to do so. Addressing the health care needs and health care

						quality of rural populations must be part of the design, development, and performance monitoring of ACOs of the future.
Peiris 2016[44]	ACO, NS	Yes	Descriptive	2012-2015	ACO practices	Compared to non-commercial ACOs, commercial ACOs tended to have a higher degree of engagement in disease monitoring activities, have taken action in the areas of care processes, unnecessary hospitalizations, and specialist referral processes. More commercial ACOs were investing in processes to reduce emergency department use and taking steps to reduce overuse of health services identified by Choosing Wisely as having low value.
Phipps 2016[45]	ACO, NS	No	Conceptual framework	-	-	Motivating behavior change within ACOs goes beyond financial incentives. ACOs are using a broad range of motivators, including creating ways to make a greater impact on patients and opportunities to be a more effective physician, such as mastery and social purpose.
Resnick 2018a[46]	MSSP	Yes	Quasi-experimental	2007-2010	Adult beneficiaries	Breast cancer screening rates declined in both ACO and non-ACO groups in the post period, with the most pronounced decline among elderly women; the decline in younger women was minimal. Slight increase in colorectal cancer screening overall in both groups, particularly among younger individuals; absolute rates remained below those for breast cancer screening. Prostate cancer screening declined in both ACO and non-ACO groups, with larger decline in the ACO group. Overall, ACO enrollment was associated with more appropriate breast and colorectal screening, although the magnitude of the observed ACO effect is modest in the early ACO experience.
Resnick 2018b[47]	ACO, NS	No	Retrospective cohort	2015	Physicians	Few surgeons participate in ACOs and considerable variation exists across practices. Factors associated with participation include surgeon specialty, practicing within a group practice or an integrated system.
Richards 2018[48]	ACO, NS	No	Retrospective cohort	2015	Physicians	Horizontal and vertical integration strongly influences measures of physician concentration; however, ACOs have limited impact overall. ACOs are often present in competitive markets, and only in a minority of these markets do ACOs substantively increase physician concentration.
Rutledge 2019[49]	Medicaid ACO	Yes	Quasi-experimental Mixed methods	2019	Adult beneficiaries	Medicaid ACOs were associated with improvements in use, quality, and expenditures, including statistically significant reductions in emergency department visits. Only Vermont's ACO demonstrated slower growth in total Medicaid expenditures (no changes in other states – namely, Maine, Massachusetts, and Minnesota).

Ryan 2018[50]	MSSP, Pioneer	Yes	Quasi-experimental	2008-2015	Hospitals	Significant reductions in 30-day readmissions for patients with acute myocardial infarction, heart failure, and pneumonia.
Schur 2017[51]	MSSP, Pioneer, ASM	No	Descriptive	2014-2015	Physicians	Many participating physicians' views are not aligned with ACO goals. Physicians are divided as to whether the ACO model is effective. Most of them view the local health care environment as moving away from fee-for-service payment alone. However, these physicians seem largely comfortable with their own ability to adapt to the shift toward value-based payment and may not view the ACO model as necessary to their success in doing so.
Schwartz 2015[52]	Pioneer	Yes	Quasi-experimental	2009-2012	Adult beneficiaries	Reduced utilization of low-value services (low to no clinical benefit).<br><br>Reduced spending on low-value services.
Shortell 2015[53]	ACO, NS	No	Mixed methods	2012-2014	ACO practices	Greater ACO use of PAE activities at the point-of-care may influence more positive leadership perceptions of the impact of PAE investments on ACO costs, quality, and outcomes of care. Important practices associated with greater PAE include high-level leadership commitment, goal-setting supported by adequate resources, extensive provider training and use of inter-disciplinary care teams, and frequent monitoring and reporting on progress.
Song 2011[54]	AQC	Yes	Quasi-experimental	2006-2009	Adult beneficiaries	Modest slowing of spending growth in AQC system. Savings driven by shifts in outpatient care toward facilities with lower fees; from lower expenditures for procedures, imaging, and testing; and from a reduction in spending for enrollees with the highest expected spending. Improvement in chronic disease management and pediatric care quality indicators, but not adult preventive care.
Song 2012[55]	AQC	Yes	Quasi-experimental	2006-2010	Adult beneficiaries	Slowing in the rate of spending in the AQC group. Savings were accounted for by lower prices achieved through shifting procedures, imaging, and tests to facilities with lower fees, as well as reduced utilization among some groups. Quality of care also improved compared to control organizations, with chronic care management, adult preventive care, and pediatric care within the contracting groups improving more in year 2 than in year 1.
Stapleton 2018[56]	ACO, NS	No	Brief report	-	Surgeons	Surgeons have an active role to play in ACOs, particularly in terms of controlling costs. They should be involved in setting quality standards for ACOs, determining appropriateness of care, and adjusting for patient risk.

Stuart 2017[57]	AQC	Yes	Quasi-experimental	2006-2011	Pediatric and adult beneficiaries	With behavioural risk: no change in substance-use disorder (SUD) service utilization, SUD spending, or SUD performance metrics.<br><br>Without behavioural risk: increase in SUD services, reduction in SUD medication use, mixed SUD performance metrics changes.
Trainor 2016[58]	Pioneer	No	Qualitative	2016	Clinical pharmacists	Few ACO practices directly involve or contract pharmacists. Pharmacists perform medication therapy management, education of patients about proper medication use, polypharmacy management, transitional care, and medication adherence and reminder programs. This may be especially helpful for high-cost patients. Next Generation ACO models are seeking to more formally integrate pharmacists in their teams. Barriers may include lack of explicit incentives for involving pharmacists in ACOs.
Trombley 2019[59]	AIM	Yes	Quasi-experimental	2013-2016	Adult beneficiaries	Provider participation in AIM was associated with a differential reduction in total Medicare spending per beneficiary per month, relative to the comparison group (beneficiaries residing in ACO markets but were served primarily by non-ACO providers). Decreases in the number of hospitalizations and use of institutional post-acute care contributed to the observed reduction in overall spending.
Wan 2014[60]	MSSP, Pioneer, ASM	Yes	Quasi-experimental	2012	Healthcare managers	When the number of perceived benefits is greater than the number of perceived barriers, health care managers are more likely to reveal a stronger commitment to develop a strategic plan for ACO adoption. Healthcare managers who perceived their organizations as lacking leadership support or commitment, financial incentives, and legal and regulatory support to ACO adoption were less willing to participate in ACOs in the future.
Winblad 2017[61]	MSSP	Yes	Quasi-experimental	2007-2013	Adults discharged from SNF	Reduction in 30-day, 1-3-day (premature-discharge indicator), and 4-30-day (SNF-quality indicator) rehospitalization.
Wright 2017[62]	Patient-Centered Shared Savings Program	No	Descriptive	2012-2014	Nurse practitioners	Nurse practitioners in PCSSP have met or exceeded the minimum scores for 29 quality metrics along with a demonstrated cost-savings in the first 2 years of the program. Hospitalization rates for NP-managed patients are among the lowest in the state. Cost of care for NP-managed patients is $66.85 less per member per month than the participating physician-managed patients.
Zhang 2017[63]	Pioneer	Yes	Quasi-experimental	2011-2012	Adult beneficiaries	Savings in overall Medicare Part A and B spending. No difference in Part D (drug) spending, total prescriptions filled, and proportion of brand-name drugs.

Accountable Care Organization, ACO; Accountable Care Units, ACU; Advanced Savings Model, ASM; ACO Investment Model, AIM; Alternative Quality Contract, AQC; Attention deficit hyperactivity disorder, ADHD; Coordinated Care Organization, CCO; Medicare Shared Savings Program, MSSP; Not specified, NS; Rural Health Clinic, RHC; Skilled Nursing Facilities, SNF

References

1. American Society of Consultant Pharmacists (ASCP). (2014). A new opportunity for pharmacists: Accountable Care Organizations. ASCP Reports. Consult Pharm, 29(9), 570. doi: 10.4140/TCP.n.2014.570.
2. Bagwell, M.T., Bushy, A., Ortiz, J. (2017). Accountable Care Organization Implementation Experiences and Rural Participation. J Nurs Admin, 47(1), 30-34.
3. Barry C.L., Stuart E.A., Donohue J.M., Greenfield S.F., Kouri E., Duckworth K., Song Z., Mechanic R.E., Chernew M.E., Huskamp H.A. (2015). The Early Impact Of The 'Alternative Quality Contract' On Mental Health Service Use And Spending In Massachusetts. Health Affairs 12:2077-2085.
4. Borza, T., Oerline M.K., Skolarus, T.A., et al. (2019). Association Between Hospital Participation in Medicare Shared Savings Program Accountable Care Organizations and Readmission Following Major Surgery. Annals of Surgery, 269(5), 873-878
5. Busch, A. B., Huskamp, H. A., & McWilliams, M. (2016). Early efforts by Medicare accountable care organizations have limited effect on mental illness care and management. Health Affairs, 35(7), 1247-1256.
6. Castle, B.W., Shapiro, S.E. (2016). Accountable Care Units: A disruptive innovation in acute care delivery. Nurs Admin, 40(1), 14-23.
7. Chien, A.T., Song, Z., Chernew, M.E., Landon, B.E., McNeil, B.J., Safran, D.G., Schuster, M.A. (2014). Two-year impact of the Alternative Quality Contract on pediatric health care quality and spending. Pediatrics, 133(1), 96-104.
8. Christensen, E. W., & Payne, N. R. (2016a). Effect of attribution length on the use and cost of health care for a pediatric Medicaid accountable care organization. JAMA Pediatrics, 170(2), 148-154.
9. Christensen, E. W., & Payne, N. R. (2016b). Pediatric inpatient readmissions in an accountable care organization. The Journal of Pediatrics, 170, 113-119.
10. Chukmaitov, A., Harless, D.W., Bazzoli, G.J., Muhlestein, D.B. (2018). Preventable Hospital Admissions and 30-Day All-Cause Readmissions: Does Hospital Participation in Accountable Care Organizations Improve Quality of Care? Am J Medical Quality, 1-9.
11. Colla, C. H., Lewis, V. A., Kao, L. S., O'Malley, J., Chang, C. H., & Fisher, E. S. (2016). Association between Medicare accountable care organization implementation and spending among clinically vulnerable beneficiaries. JAMA Internal Medicine, 176(8), 1167-1175.

12. Colla, C.H., Lewis, V.A., Stachowski, C., Usadi, B., Gottlieb, D.J., Bynum, J.P.W. (2019). Changes in Use of Postacute Care Associated With Accountable Care Organizations in Hip Fracture, Stroke, and Pneumonia Hospitalized Cohorts. Medical Care, 57(6), 444-452
13. Comfort, L.N, Shortell, S.M., Rodriguez, H.P., Colla, C.H. (2018). Medicare accountable care organizations of diverse structures achieve comparable quality and cost performance. Health Services Research Educational Trust, doi: 10.1111/1475-6773.12829.
14. D'Aunno, T., Broffman, L., Sparer, M., Kumar, S.R. (2018). Factors that distinguish high-performing accountable care organizations in the Medicare Shared Savings Program. Health Services Research Educational Trust, doi: 10.1111/1475-6773.12642.
15. Dupree, J.M., Patel, K., Singer, S.J., West, M., Zinner, M.J., & Weissman, J.S. (2014). Attention to surgeons and surgical care is largely missing from early Medicare Accountable Care Organizations. Health Affairs, 33(6), 972-979.
16. United States Government Accountability Office. (2015). Results from the First Two Years of the Pioneer Accountable Care Organization Model. Report to the Ranking Member, Committee on Ways and Means, House of Representatives. Available at: https://www.gao.gov/assets/670/669782.pdf
17. Geyer B.C., Peak D.A., Velmahos G.C., Gates J.D., Michaud Y., Petrovick L., Lee J., Yun B.J., White B.A., Raja A.S. (2016). Cost savings associated with transfer of trauma patients within an accountable care organization. American Journal of Emergency Medicine 34:455-458.
18. Gittel, J.H., Beswick, J., Goldmann, D., Wallack, S.S. (2015). Teamwork methods for accountable care: Relational coordination and TeamSTEPPS. Health Care Manage Rev, 40(2), 116-125.
19. L&M Policy Research. (2015). Pioneer ACO Evaluation Findings from Performance Years One and Two. Report prepared for Centers for Medicare & Medicaid Services. Available at: https://www.abtassociates.com/insights/publications/report/evaluation-of-cmmi-accountable-care-organization-initiatives-pioneer. Last accessed: September 30, 2018
20. Green, L. on behalf of L&M Policy Research. (2016). Evaluation of CMMI Accountable Care Organization Initiatives: Advance Payment ACO Final Report. Available at: https://innovation.cms.gov/files/reports/advpayaco-fnevalrpt.pdf
21. Herrell L.A., Norton E.C., Hawken S.R., Ye Z., Hollenbeck B.K., Miller D.C. (2016). Early Impact of Medicare Accountable Care Organizations on Cancer Surgery Outcomes. Cancer. 10.1002/cncr.30111
22. Hewner S., Casucci S., Castner J. (2016). The roles of chronic disease complexity, health system integration, and care management in post-discharge healthcare utilization in a low-income population. Research in Nursing & Health 39:215-228.
23. Hofler, R.A. & Ortiz, J. (2016). Costs of accountable care organization participation for primary care providers: early stage results. BMC Health Services Research, 16, 315, doi: 10.1186/s12913-016-1556-6.

24. Hsu, J., Vogeli, C., Price, M., Brand, R., Chernew, M.E., Mohta, N., Chaguturu, S.K. (2017). Substantial Physician Turnover And Beneficiary'Churn' In A Large Medicare Pioneer ACO. Health Affairs, Chevy Chase, 36(4), 640-648
25. Huskamp H.A., Greenfield S.F., Stuart E.A., Donahue J.M., Duckworth K., Kouri E.M., Song Z., Chernew M.E., Barry C.L. (2016). Effects of Global Payment and Accountable Care on Tobacco Cessation Service Use: An Observational Study. Journal of General Internal Medicine 31(10):1134-1140.
26. Joseph, T., Hale, G.M, Eltaki, S.M., Prados, Y., Jones, R., Seamon, M.J., Moreau, C., Gernant, S.A. (2017). Integration Strategies of Pharmacists in Primary Care-Based Accountable Care Organizations: A Report from the Accountable Care Organization Research Network, Services, and Education. J Manag Care Spec Pharm, 23(5), 541-48.
27. Joyce N.R, Huskamp H.A, Hadland S.E., Donohue J.M., Greenfield S.F., Stuart E.A., Barry C.L. (2017). The Alternative Quality Contract: Impact on Service Use and Spending for Children With ADHD. Psychiatric Services 68(12):1210-1212.
28. Kelleher, K. J., Cooper, J., Deans, K., Carr, P., Brilli, R. J., Allen, S., & Gardner, W. (2015). Cost saving and quality of care in a pediatric accountable care organization. Pediatrics, 135(3), e582-e589.
29. Kleiner, S.A., Ludwinski, D., White, D.W. (2017). Antitrust and Accountable Care Organizations: Observations for the Physician Market. Medical Care Research and Review, 74(1), 97-108.
30. Lowell, K.H. (2018). Next Generation Accountable Care Organization (NGACO) Model Evaluation. NORC at the University of Chicago. Retrieved from https://innovation.cms.gov/Files/reports/nextgenaco-firstannrpt.pdf
31. McConnell J., Renfro S., Chan B.K>S., Meath T.H.A., Mendelson A., Cohen D., Waxmonsky J., McCarthy D., Wallace N., Lindrooth R.C. (2017). Early Performance in Medicaid Accountable Care Organizations: A Comparison of Oregon and Colorado. JAMA Internal Medicine 177(4):538-545.
32. McWilliams, J.M., Landon, B.E., Chernew, M.E. (2013). Changes in Health Care Spending and Quality for Medicare Beneficiaries Associated With a Commercial ACO Contract. JAMA, 310(8), 829-836.
33. McWilliams, J.M., Landon, B.E., Chernew, M., Zaslavsky, A.M. (2014). Changes in Patients' Experiences in Medicare Accountable Care Organizations. N Engl J Med, 371, 1715-24.
34. McWilliams, J. M., Hatfield, L. A., Chernew, M. E., Landon, B. E., & Schwartz, A. L. (2016). Early performance of accountable care organizations in Medicare. New England Journal of Medicine, 374(24), 2357-2366.
35. McWilliams, J. M., Gilstrap, L. G., Stevenson, D. G., Chernew, M. E., Huskamp, H. A., & Grabowski, D. C. (2017). Changes in postacute care in the Medicare Shared Savings Program. JAMA Internal Medicine, 177(4), 518-526.

36. McWilliams, J.M. (2018a). Evaluation of the accountable care organization investment model: AIM impacts in the first performance year (Report prepared for Centers for Medicare & Medicaid Services). Retrieved from https://innovation.cms.gov/Files/reports/aim-firstannrpt.pdf
37. McWilliams, J.M., Hatfield, L.A., Landon, B.E., Hamed, P., Chernew, M.E. (2018b). Medicare Spending after 3 Years of the Medicare Shared Savings Program. N Engl J Med, 379, 1139-49.
38. Muhlestein D., Tu T., de Lisle K., Merrill T. (2016). Hospital Participation in ACOs Associated With Other Value-Based Program Improvement. American Journal of Managed Care 22(7):e241-e248.
39. Narayan, A. K., Harvey, S. C., Durand, D. J. (2017). Impact of Medicare shared savings program accountable care organizations at screening mammography: A retrospective cohort study. Radiology, 282(2), 437-442.
40. Neprash, H.T., Chernew, M.E., McWilliams, J.M. (2017). Little Evidence Exists To Support The Expectation That Providers Would Consolidate To Enter New Payment Models. Health Affairs, 36(2), 346-354.
41. Noble, D.J., Greenhalgh, T., Casalino, L.P. (2014). Improving population health one person at a time? Accountable care organisations: perceptions of population health—a qualitative interview study. BMJ Open, 4:e004665.
42. Nyweide, D. J, Lee, W., Cuerdon, T. T., Pham, H. H., Cox, M., Rajkumar, R., & Conway, P.H. (2015). Association of pioneer accountable care organizations vs traditional Medicare fee for service with spending, utilization, and patient experience. JAMA, 213(21), 2152-2161.
43. Ortiz, J., Hofler, R.A., Lin, Y.L., Berzon, R. Participation of Rural Health Care Providers in Accountable Care Organizations: Early Indications. (2015). The Health Care Manager, 34(3), 255-264.
44. Peiris D., Phipps-Taylor M.C., Stachowski C.A., Kao L.S., Shortell S.M., Lewis V., Rosenthal M.B., Colla C.H. (2016). ACOs Holding Commercial Contracts Are Larger And More Efficient Than Noncommercial ACOs. Health Affairs 35(10):1849-1856.
45. Phipps-Taylor, M. & Shortell, S. (2016). More Than Money: Motivating Physician Behavior Change in Accountable Care Organizations. The Milbank Quarterly, 94(4), 832-861.
46. Resnick, M.J., Graves, A.J., Thapa, S., Gambrel, R., Tyson, M.D., Lee, D., Buntin, M.B., Penson, D.F. (2018a). Medicare Accountable Care Organization Enrollment and Appropriateness of Cancer Screening. JAMA Internal Med, 178(5), 648-654.
47. Resnick, M.J., Graves, A.J., Buntin, M.B., Richards, M.R., Penson, D.F. (2018b). Surgeon Participation in Early Accountable Care Organizations. Ann Surg, 268, 401-407.
48. Richards, M.R., Smith, C.T., Graves, A.J., Buntin, M.B., & Resnick, M.J. (2018). Physician Competition in the Era of Accountable Care Organizations. Health Research and Educational Trust, doi: 0.1111/1475-6773.12690.

49. Rutledge, R.I., Romaire, M.A., Hersey C.L., Parish, W.J., Kissam, S.M., Lloyd, J.T. (2019). Medicaid Accountable Care Organizations in Four States: Implementation and Early Impacts. Milbank Quarterly, 97(2), 583-619
50. Ryan, A.M., Krinsky, S., Adler-Milstein, J., Damberg, C.L., Maurer, K.A., Hollingworth, J.M. (2017). Association Between Hospitals' Engagement in Value-Based Reforms and Readmission Reduction in the Hospital Readmission Reduction Program. JAMA Internal Med, 177(6), 862-868.
51. Schur, C.L. & Sutton, J.P. (2017). Physicians In Medicare ACOs Offer Mixed Views Of Model For HealthCare Cost And Quality. Health Affairs, 36(4), 649-654.
52. Schawrtz A.L., Chernew M.E., Landon B.E., McWilliams J.M. (2015). Changes in Low-Value Services in Year 1 of the Medicare Pioneer Accountable Care Organization Program. JAMA Internal Medicine 175(11):1815-1825.
53. Shortell S.M., Sehgal N.J., Bibi S., Ramsay P.P., Neuhauser L., Colla C.H., Lewis V.A. (2015). An early assessment of Accountable Care Organizations' efforts to engage patients and their families. Medical Care Research and Review 72(5):580-604.
54. Song, Z., Safran, D.G., Landon, B.E., He, Y., Ellis, R.P., Mechanic, R.E., Day, M.P., Chernew, M.E., 2011. Health care spending and quality in year 1 of the alternative quality contract. N. Engl. J. Med. 365, 909–18. https://doi.org/10.1056/NEJMsa1101416
55. Song, Z., Safran, D.G., Landon, B.E., Landrum, M.B., He, Y., Mechanic, R.E., Day, M.P., Chernew, M.E. (2012). The 'Alternative Quality Contract ,'Based On A Global Budget, Lowered Medical Spending And Improved Quality. Health Affairs, 31(8), 1885-1894.
56. Stapleton, S.M., Chang, D.C., Rattner, D.W., Ferris, T.G. (2018). Along for the Ride? Surgeon Participation in Accountable Care Organizations.
57. Stuart E.A., Barry C.L., Donohue J.M., Greenfield S.F., Duckworth K., Song Z., Kouri E.M., Ebnesajjad C., Mechanic R. (2017). Effects of Accountable Care and Payment Reform on Substance Use Disorder Treatment: Evidence from the Initial Three Years of the Alternative Quality Contract. Addiction 112(1):124-133.
58. Trainor, K. (2016). Pharmacits' participation in ACO has room to grow. Am J Health-Syst Pharm, 73(9), e118-e119.
59. Trombley, M.J., Fout, B., Brodsky, S., McWilliams, M.J., Nyweide, D.J., Morefield, B. (2019). Early Effects of an Accountable Care Organization Model for Underserved Areas. NEJM 381(6), 543-551
60. Wan, T.H., Demachkie Mastri, M., Ortiz, J., Blosson, Y. (2014). Willingness to Participate in Accountable Care Organizations: Health Care Managers' Perspective. The Health Care Manager, 33(1), 64-74.
61. Winblad, U., Mor, V., McHugh, J. P., & Rahman, M. (2017). ACO-affiliated hospitals reduced rehospitalizations from skilled nursing facilities faster than other hospitals. Health Affairs, 36(1), 67-73.

62. Wright, W.L. (2017). New Hampshire nurse practitioners take the lead in forming an Accountable Care Organization. Nurs Admin Q, 41(1), 39-47.
63. Zhang, Y., Caines, K. J., & Powers, C. A. (2017). Evaluating the effects of pioneer accountable care organizations on Medicare part d drug spending and utilization. Medical Care, 55(5), 470-475.
